# Are persistent delusions in schizophrenia associated with aberrant salience?

**DOI:** 10.1016/j.scog.2016.04.002

**Published:** 2016-05-18

**Authors:** Rafeef Abboud, Jonathan P. Roiser, Hind Khalifeh, Sheila Ali, Isobel Harrison, Helen T. Killaspy, Eileen M. Joyce

**Affiliations:** a Institute of Cognitive Neuroscience, University College London, 17 Queen Square, London, WC1N 3AZ, UK; b Camden and Islington NHS Foundation Trust, St Pancras Hospital, 4 St. Pancras Way, London, NW1 0PE, UK; c Division of Psychiatry, University College London, 6th Floor, Maple House, 149 Tottenham Court Road, London, W1T 7NF, UK; d Institute of Psychiatry, Psychology and Neuroscience, Kings College London, De Crespigny Park, London, SE5 8AF; e Institute of Neurology, University College London, Queen Square, London, WC1N 3BG, UK

**Keywords:** Schizophrenia, Psychosis, Delusions, Reinforcement, Behaviour

## Abstract

**Objective:**

It has been suggested that positive psychotic symptoms reflect ‘aberrant salience’. Previously we provided support for this hypothesis in first-episode schizophrenia patients, demonstrating that delusional symptoms were associated with aberrant reward processing, indexed by the Salience Attribution Test (SAT). Here we tested whether salience processing is abnormal in schizophrenia patients with long-standing treatment-refractory persistent delusions (TRS).

**Method:**

Eighteen medicated TRS patients and 31 healthy volunteers completed the SAT, on which participants made a speeded response to earn money in the presence of cues. Each cue comprised two visual dimensions, colour and form. Reinforcement probability varied over one of these dimensions (task-relevant), but not the other (task-irrelevant).

**Results:**

Participants responded significantly faster on high-probability relative to low-probability trials, representing implicit adaptive salience; this effect was intact in TRS patients. By contrast, TRS patients were impaired on the explicit adaptive salience measure, rating high-probability stimuli less likely to be associated with reward than controls. There was little evidence for elevated aberrant salience in the TRS group.

**Conclusion:**

These findings do not support the hypothesis that persistent delusions are related to aberrant motivational salience processing in TRS patients. However, they do support the view that patients with schizophrenia have impaired reward learning.

## 1 Introduction

Advances in understanding the role of dopamine in reward learning support the hypothesis that psychotic symptoms reflect the formation of abnormal stimulus-reinforcement associations (Kapur, 2003). It is well established that mesolimbic dopamine transmission is involved in predicting rewarding events and coding outcome expectancies (Berridge, 2007, Wise, 2004). Stimuli that are repeatedly paired with a reward, termed conditioned stimuli (CS +), are able to elicit phasic dopamine firing in the midbrain when presented alone, while unconditioned stimuli (CS −) that do not predict reward do not elicit such a response (Schultz et al., 1997). It has been shown, in humans and in animals, that presentation of a CS + leads to increased response vigour compared to the presentation of a CS − (Cools et al., 2008, Roiser et al., 2006, Talmi et al., 2008, Wyvell and Berridge, 2000), an effect that is modulated by ventral striatal dopamine (Wyvell and Berridge, 2000). This effect has been interpreted as reflecting ‘motivational salience’ (Berridge and Robinson, 1998, Milstein and Dorris, 2007).

A number of theorists have suggested that the formation of abnormal stimulus-reinforcement associations in schizophrenia might be related to dysregulated dopamine transmission in the ventral striatum (Gray et al., 1991, King et al., 1984, Shaner, 1999, Snyder, 1976). Disrupted motivational salience processing has been proposed to contribute to the development of abnormal beliefs in psychotic disorders, and in particular in patients with schizophrenia (Corlett et al., 2007, Hemsley, 1993, King et al., 1984, Maher, 1974, Miller, 1976, Shaner, 1999). Kapur (2003) proposed the aberrant salience hypothesis of psychosis, suggesting that the positive symptoms of schizophrenia may arise from the aberrant assignment of salience to external objects and internal representations, via context-independent stimulus-reinforcement signalling, driven by chaotic dopamine neuron firing (Seeman and Kapur, 2000). This model also proposes that antipsychotic medications contribute to the resolution of positive symptoms by attenuating aberrant motivational salience, via blockade of the dopamine D2 receptor (Kapur, 2003). However, a necessary corollary of this is that antipsychotic medication will also necessarily attenuate adaptive (appropriate) motivational salience which may result in negative side-effects related to loss of motivation, such as apathy and anhedonia.

Consistent with this model, numerous studies have demonstrated reinforcement learning deficits in schizophrenia (Gold et al., 2008, Murray et al., 2008b, Waltz et al., 2007, Waltz and Gold, 2007), although the patients included in these studies were mostly receiving medication; important because anti-psychotic medication is known to disrupt reward processing in healthy volunteers (Pessiglione et al., 2006). Findings of abnormal haemodynamic response patterns in regions strongly innervated by dopamine during reward processing in patients with psychosis, including in unmedicated patients, provide convergent support for the aberrant salience hypothesis (Jensen et al., 2008, Juckel et al., 2006, Murray et al., 2008a); though interestingly a recent longitudinal neuroimaging study demonstrated relatively normalised haemodynamic responses during the anticipation of uncertain rewards following 6 weeks' treatment with an atypical antipsychotic (amisulpride) in initially treatment-naïve patients (Nielsen et al., 2012).

Roiser et al. (2009) provided the first evidence linking aberrant reward learning with delusions, as predicted by the aberrant salience hypothesis, using a novel behavioural paradigm, the Salience Attribution Test (SAT), designed specifically to assess processing pertaining to irrelevant stimuli. First-episode schizophrenia patients with delusions scored higher on the SAT “explicit aberrant salience” measure (task-irrelevant reward learning) than those without such symptoms. Consistent with the above-mentioned studies they also identified impaired “adaptive salience” (task-relevant reward learning) in these predominantly medicated patients with first-episode schizophrenia. More recently, Roiser et al. (2013) reported elevated aberrant salience in “prodromal” individuals, who are at high risk of the development of psychosis because they experience attenuated psychotic symptoms (for example, suspicious ideas or unusual perceptual experiences), but do not (yet) meet criteria for psychosis. Interestingly, the degree of aberrant salience measured by the SAT correlated positively with delusion-like symptoms in that sample.

Importantly, Roiser et al. (2009) did not demonstrate a significant difference in aberrant salience between healthy volunteers and patients with first-episode schizophrenia. However, that study included a heterogeneous sample with only 65% of patients actually reporting delusions at the time of testing, with moderate severity. If a relationship between aberrant salience and delusions does indeed exist, it was anticipated that selecting a symptomatically homogeneous sample of patients with severe delusions would reduce variability and thereby increase the chance of finding a difference between patients with schizophrenia and healthy volunteers. Hence, the patients recruited for the present study were receiving medication but still exhibiting florid positive psychotic symptoms, specifically delusions.

### 1.1 Aims of the study

The aim of the current study was to test whether motivational salience processing was abnormal in schizophrenia patients experiencing persistent delusions. On the basis of the aberrant salience hypothesis we made the following predictions:

1. Patients experiencing persistent delusions would exhibit greater aberrant salience than healthy volunteers, reflecting a pathological neurobiological mechanism maintaining their delusions;
2. Patients experiencing persistent delusions would exhibit reduced adaptive salience relative to healthy volunteers, reflecting a corollary of anti-psychotic medication.

## 2 Methods

### 2.1 Participants

Eighteen patients with schizophrenia who had long-standing treatment-refractory persistent delusions (TRS) were recruited from five mental health rehabilitation units within an inner city UK National Health Service Trust. Eligible patients were invited to participate following review of their clinical notes and discussion with their psychiatrist. This Trust serves a population with one of the highest levels of psychiatric morbidity in the UK and provides a full range of inpatient and community based services. People receiving inpatient mental health rehabilitation (who were the target of recruitment for the present study) are those with especially complex psychosis (treatment resistant positive and/or negative symptoms, often with mental and physical health comorbidities) who require specialist treatment due to the severity of their illness.

Patients were recruited if they were aged 18–65 years. The inclusion criteria were a DSM-IV diagnosis of schizophrenia and the presence of persistent delusions despite adequate treatment with at least two antipsychotic drugs at therapeutic doses for at least 6 weeks (Table 1). Patients of these rehabilitation services had been in contact with mental health services from 15 to 30 years, with a mean of five previous admissions to hospital and had spent between five months and five years in their current unit (Killaspy et al., 2008). Symptom type and severity were assessed in patients at the time of testing using the Scales for the Assessment of Positive Symptoms – SAPS (Andreasen, 1983) and Negative Symptoms – SANS (Andreasen, 1981), the Calgary Depression Rating Scale for Schizophrenia – CDRSS (Addington et al., 1990) and the Young Mania Scale – YMRS (Young et al., 1978).

**Table 1:** Demographic, personality and clinical measures.

	Controls (*N* = 31)	*N*	Patients (*N* = 18)	*N*	Statistic
**Gender (male/female)**	21/10	31	14/4	18	χ ^2^(1) = 0.562, *P* = 0.453
**Age, years**	36.80 (11.32)	31	41.82 (12.25)	17*	t(35) = 1.426, *P* = 0.160
**Estimated full scale IQ (WTAR or NART)**	96.55 (9.14)	31	91.13 (13.38)	15**	t(33) = 1.481, *P* = 0.146
**O-LIFE Unusual Experiences**	2.80 (3.32)	31	-	-	-
**O-LIFE Cognitive Disorganization**	3.48 (2.91)	31	-	-	-
**O-LIFE Introvertive Anhedonia**	1.67 (1.75)	31	-	-	-
**O-LIFE Impulsive Nonconformity**	2.45 (2.12)	31	-	-	-
**SAPS global**	-	-	7.17 (2.60)	18	-
**SANS global**	-	-	5.72 (3.71)	18	-
**Delusions - global severity**	-	-	3.94 (0.73)	18	-
**Hallucinations - global severity**	-	-	2.22 (2.05)	18	-
**Antipsychotic intake (% of maximum BNF dose)**	-	-	69.3 (43.9)	18	-

Values represent means (standard deviations). Abbreviations: IQ, intelligence quotient; WTAR, Wechsler test of adult reading; NART, National Adult Reading Test; O-LIFE, Oxford-Liverpool Inventory of Feelings and Experiences; SAPS, Scale for the Assessment of Positive Symptoms; SANS, Scale for the Assessment of Negative Symptoms; BNF, British National Formulary. * denotes age data missing from one patient. ** denotes IQ data missing from 3 patients: one patient was dyslexic; one patient did not have English as their first language; and one patient's data were not recorded. The proportion of participants tested with the WTAR (controls: *N* = 21; patients: *N* = 7) and the NART (controls: *N* = 10; patients: *N* = 8) did not differ significantly between the groups (χ^2^_1_ = 1.885, *P* = 0.170).

All patients were medicated at the time of testing. Total antipsychotic medication load was computed as a percentage of the maximum recommended British National Formulary (BNF: www.bnf.org) dose, summing percentages for different antipsychotics where appropriate. Eight patients were taking one atypical antipsychotic, two were taking two atypical antipsychotics, three were taking one atypical antipsychotic and one mood stabiliser (e.g. sodium valproate), four were taking two atypical antipsychotics and one mood stabiliser, and one patient was taking one atypical antipsychotic, one typical antipsychotic (haloperidol), and one mood stabiliser. In total eight patients were taking clozapine.

Patients were compared with thirty-one healthy volunteers, recruited via advertisement. Exclusion criteria were: known psychiatric or neurological disorder; medical disorder likely to lead to cognitive impairment; intelligence quotient (IQ) < 70; and recent illicit substance use. The absence of axis-I psychopathology and alcohol- or substance-abuse/dependence was confirmed with the Mini International Neuropsychiatric Inventory (Sheehan et al., 1998). Dimensions of schizotypy were measured in the healthy volunteers using the short form of the Oxford-Liverpool Inventory of Feelings and Experiences schizotypy questionnaire – O-LIFE (Mason et al., 2005). Ethical approval was obtained from the Ealing & West London Mental Health Trust Research Ethics Committee. All participants provided written informed consent, and were compensated £40 for their time and travel expenses.

### 2.2 Experimental paradigm – Salience Attribution Test (SAT)

The SAT is a speeded reaction time task in which participants respond to a probe (a black square) following presentation of a cue in order to earn money (Roiser et al., 2009). A full description is provided in the Supplementary Online Materials.

Participants completed two blocks of 64 trials, where money was available on 50% of trials. On each trial, the likelihood that money was available was signalled by one of four cues that appeared at the top and bottom of the screen just before the onset of the probe (the black square). Four different types of cues were used: red animals; blue animals; red household objects and blue household objects, which varied across two dimensions - colour (blue or red) and form (animal or household object). One of the dimensions (for example, colour) was *task-relevant* so that one level of the dimension (for example blue) was reinforced on 28 out of 32 (87.5%) of the trials, while only 4 out of 32 (12.5%) trials of the other level were reinforced. The other dimension, in this example form, was *task-irrelevant*, so that 16 out of 32 (50%) of both levels (animals and household objects) were reinforced. Four different versions of the SAT were used, each with a different stimulus feature (blue, red, animal or household object) reinforced with high probability. Each participant was administered the same version (i.e. with identical cue-reinforcement contingencies) for both blocks.

### 2.3 Other cognitive measures

Verbal IQ was estimated using the Wechsler Test of Adult reading – WTAR (Wechsler, 2001) or the National Adult Reading Test – NART (Nelson, 1982). The forwards and backwards digit span was used to test whether patients with schizophrenia showed an (expected) impairment in working memory (Wechsler, 1981).

### 2.4 Statistical analysis

Data were analysed using the Statistical Package for Social Sciences, version 16 (SPSS Inc., Chicago, IL, USA). Full details are provided in the Supplementary Online Materials.

At alpha = 0.05 (two-tailed), this study had 80% power to detect a large effect size (*d* = 0.85), which is of comparable magnitude to the increase in explicit aberrant salience we previously identified in individuals in an at-risk mental state for psychosis, not all of whom had delusion-like thoughts, relative to healthy volunteers (*d* = 0.93: Roiser et al., 2013).

## 3 Results

### 3.1 Demographic data

The groups were matched for gender, age and IQ (Table 1).

### 3.2 Digit span

Controls scored significantly higher than patients on the digit span task [main effect of group: F(1,46) = 16.616, *P* < 0.001], and scores were significantly lower in the backwards condition than the forwards condition [main effect of stage: F(1,46) = 33.965, *P* < 0.001]. The group*stage interaction was non-significant (*P* > 0.1).

### 3.3 Salience Attribution Test (SAT)

Behavioural data are presented in Table 2.

**Table 2:** Behavioural data.

Test	Measure	Controls (*N* = 31)	Patients (*N* = 18)
**Salience Attribution Test**			
**Block 1**	RT high probability (ms)*	250.09 (54.15)	351.12 (108.84)
	RT low probability (ms)*	253.93 (56.76)	367.89 (145.97)
	Implicit adaptive salience (ms)	3.84 (19.86)	16.77 (47.55)
	Implicit aberrant salience (ms)^†#^	12.80 (12.18)	34.07 (23.49)
	VAS high probability (%)^#^	54.52 (17.12)	40.14 (18.95)
	VAS low probability (%)	27.34 (17.65)	30.97 (18.31)
	Explicit adaptive salience (%)*	27.18 (26.22)	9.17 (15.46)
	Explicit aberrant salience (%)	15.08 (14.68)	10.28 (8.99)
	Premature responses*	3.42 (1.98)	5.56 (4.29)
	Omissions*	0.61 (1.38)	2.06 (2.10)
**Block 2**	RT high probability (ms)*	232.40 (43.47)	345.94 (121.28)
	RT low probability (ms)*	246.26 (48.20)	353.72 (123.06)
	Implicit adaptive salience (ms)	13.86 (24.86)	7.78 (21.67)
	Implicit aberrant salience (ms)^†^	23.01 (32.65)	19.36 (13.49)
	VAS high probability (%)^#^	59.92 (17.65)	35.00 (18.17)
	VAS low probability (%)	20.65 (16.97)	28.47 (14.83)
	Explicit adaptive salience (%)*	39.27 (26.57)	6.53 (12.64)
	Explicit aberrant salience (%)	9.92 (15.16)	10.14 (6.99)
	Premature responses*	3.10 (2.53)	4.39 (3.55)
	Omissions*	0.52 (0.96)	1.44 (2.04)

**Digit span**			
	Forwards*	8.84 (2.40)	6.41 (2.29)
	Backwards*	6.77 (2.55)	4.35 (1.41)

Values represent means (standard deviations). Abbreviations: RT, response time; VAS, Visual Analogue Scale. * Significant overall main effect of group (i.e. averaging across block and/or condition) at *P* < 0.05; † significant group-by-block interaction at *P* < 0.05; # significant simple main effect of group at *P* < 0.05. Digit span data were missing for one participant. Digit span data were missing for one TRS patient.

### 3.4 Implicit salience

#### 3.4.1 Adaptive salience

The control group responded significantly faster overall than the patient group [main effect of group: F(1,47) = 19.705, *P* < 0.001] and participants responded significantly faster on block 2 than block 1 [main effect of block: F(1,47) = 5.051, *P* = 0.029]. Participants also responded significantly faster on high- relative to low-probability reinforced trials [main effect of probability: F(1,47) = 12.222, *P* = 0.001], representing implicit adaptive salience. No interactions with group approached significance (*P* > 0.1 for all). Both groups exhibited implicit adaptive salience (see Table 1), though this effect was only marginally significant in the TRS group [controls: mean = 8.85 ms, SD = 17.34 ms, t(30) = 2.841, *P* = 0.008; patients: mean = 12.28 ms, SD = 24.87 ms, t(17) = 2.093, *P* = 0.052]. When digit span (forward) was included as a covariate in the analysis the results were unchanged.

#### 3.4.2 Aberrant salience

There was a significant main effect of group [F(1,47) = 6.236, *P* = 0.016], but this was qualified by a significant group*block interaction [F(1,47) = 5.026, *P* = 0.030]. There was no significant main effect of block [F(1,47) = 0.364, *P* > 0.1]. Post-hoc analysis revealed that the TRS group exhibited significantly greater implicit aberrant salience than controls on block 1 (*P* = 0.001), while there was no difference between the groups on block 2 (*P* = 0.805). When digit span (forward) was included as a covariate in the analysis the group*block interaction no longer achieved significance (*P* = 0.121).

### 3.5 Explicit salience

#### 3.5.1 Adaptive salience (Fig. 1)

Participants rated high-probability reinforced stimuli as significantly more likely to yield reward than low-probability reinforced stimuli [main effect of probability: F(1,47) = 54.998, *P* < 0.001]. There was a trend towards patients' ratings of reward probabilities being lower overall than controls [main effect of group: F(1,47) = 3.834, *P* = 0.056]. However, these effects were qualified by a significant probability x group interaction [F(1,47) = 20.997, *P* < 0.001]. There was also a trend towards a probability*group*block interaction [F(1,47) = 3.692, *P* = 0.061], which was not analysed further. When digit span (forward) was included as a covariate in the analysis the main effect of group no longer approached significance (*P* = 0.167).

**Fig. 1:**
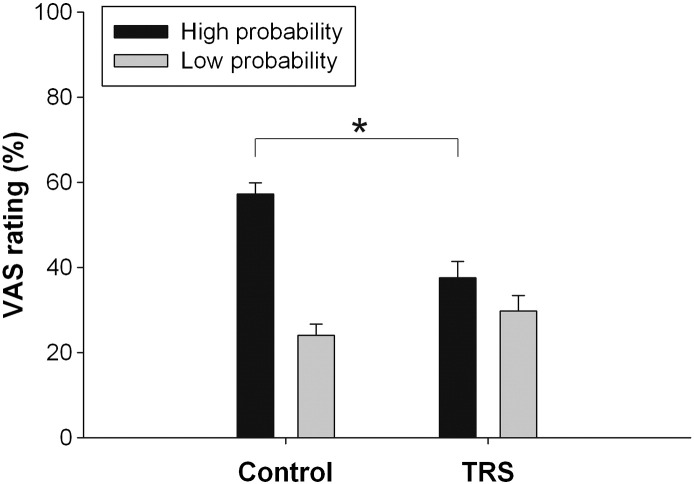
Visual analogue scale (VAS) probability ratings in schizophrenia patients with treatment-resistant delusions (TRS) and controls. Controls exhibited significantly greater explicit adaptive salience (the difference in VAS rating between high and low probability stimuli) than TRS patients [controls: mean = 33.22, SD = 21.57; patients: mean = 7.85, SD = 12.03; F(1,47) = 20.997, *P* < 0.001]. This group difference was driven by a reduction in the ratings for high probability stimuli in TRS patients (* indicates *P* < 0.001), while there was no difference between the groups for the ratings of low probability stimuli. Error bars represent standard errors of the mean.

Post-hoc analysis of the probability*group interaction revealed that the explicit adaptive salience scores were significant in each group separately, suggesting that both groups displayed some learning [controls: mean = 33.22, SD = 21.57, t(30) = 8.576, *P* < 0.001; patients: mean = 7.85, SD = 12.03, t(17) = 2.768, *P* = 0.013]. Further analysis suggested that the patients were only impaired at learning about high-probability reinforced stimuli (Fig. 1). Controls rated high probability stimuli significantly more likely to yield reward than patients (*P* < 0.001); by contrast, there was no difference between the groups for the ratings of low probability stimuli (*P* = 0.206).

#### 3.5.2 Aberrant salience

There was a trend towards a block*group interaction [F(1,47) = 3.428, *P* = 0.070], which was not analysed further. The main effects of group [F(1,47) = 0.248, *P* = 0.621] and block [F(1,47) = 1.394, *P* = 0.244] were non-significant. Mean aberrant salience scores were 10.21 (SD = 5.99) in the TRS group and 12.50 (SD = 12.15) in the controls. When digit span (forward) was included as a covariate in the analysis the block*group interaction no longer approached significance (*P* = 0.357).

### 3.6 Premature responses & omission errors

Participants showed a trend towards making fewer premature responses on the second block [F(1,47) = 3.741, *P* = 0.059], and patients made significantly more premature responses than controls [main effect of group: F(1,47) = 4.637, *P* = 0.036]. The block*group interaction was non-significant [F(1,47) = 1.202, *P* > 0.1]. When digit span (forward) was included as a covariate in the analysis the main effect of block no longer approached significance (*P* = 0.157). Patients made significantly more omission errors than the controls (*P* = 0.004), but the main effect of block was non-significant (*P* = 0.131).

### 3.7 Correlations

There were no correlations that approached significance (*P* < 0.05) between salience scores and symptom or schizotypy scores.

Across both groups explicit adaptive salience correlated significantly with IQ (*r* = 0.41, *P* = 0.005). This correlation was also significant in the TRS group alone (*r* = 0.66, *P* = 0.008). Further analysis revealed that this relationship was driven by a significant (negative) relationship between IQ and VAS ratings for low probability stimuli (all subjects: *r* = − 0.51, *P* < 0.001; controls: *r* = − 0.43, *P* = 0.015; TRS: *r* = − 0.59, *P* = 0.021), while there was no correlation between IQ and VAS ratings for high probability stimuli (*r*s < 0.1 in each group). No correlations with IQ approached significance for any other SAT measure (all *r*s < 0.3).

There was a trend towards a positive correlation between implicit adaptive salience and antipsychotic medication intake (*r* = 0.53, *P* = 0.022). There was also a trend towards a positive correlation between the number of omission errors and antipsychotic medication intake (*r* = 0.53, *P* = 0.025). The relationship with medication intake did not approach significance for any other SAT measure (all *r*s < 0.5).

## 4 Discussion

We investigated the learning of stimulus-reinforcement associations in TRS patients with chronic delusions and healthy controls using the SAT. As predicted, TRS patients exhibited reduced explicit adaptive salience relative to healthy controls. Contrary to our predictions, however, the data did not suggest reduced implicit adaptive salience or elevated aberrant salience in the TRS patients. These data do not support the hypothesis that persistent delusions in TRS patients are associated with aberrant attributions of motivational salience to external stimuli, or that antipsychotic medication causes an *implicit* motivational deficit (Kapur, 2003).

The analysis of VAS ratings revealed that participants across both groups rated high-probability-reinforced stimuli significantly more likely to yield reward than low-probability-reinforced stimuli. However, consistent with our previous study (Roiser et al., 2009), TRS patients' explicit adaptive salience scores were significantly lower than those of controls. This finding is consistent with findings from several other studies (Jensen et al., 2008, Waltz et al., 2007). This impairment in reward learning may be related to dopamine D2 receptor blockade (Cutmore and Beninger, 1990), representing an undesirable side effect of antipsychotic medication (Schooler, 1994). The adaptive salience scores were, nevertheless, significant in each group separately, suggesting that learning was not completely abolished in the TRS group.

Across both groups, participants were significantly faster at responding to high- relative to low-probability-reinforced stimuli. TRS patients were just as able to speed responses on high probability trials as controls; implicit adaptive salience scores were even numerically higher in this group and showed a positive relationship with antipsychotic medication load (though the latter finding did not survive the more stringent significance threshold we adopted for the correlational analyses). These surprising findings contradict our second prediction, and raise the possibility that although implicit and explicit adaptive salience correlate in normative samples (for example, Schmidt and Roiser (2009)), they may have different neurobiological substrates. The control group responded significantly faster on the SAT than the TRS group overall, consistent with numerous other studies (Prouteau et al., 2004).

The finding of preserved implicit adaptive salience in TRS patients is consistent with previous research suggesting that poor decision-making in schizophrenia can occur in the context of spared implicit reinforcement learning. For example, Heerey et al. (2008) reported normal sensitivity to reward in participants with schizophrenia using a signal detection approach, but differences in choice patterns on a decision-making task. Relatively intact implicit responding, in conjunction with impaired explicit responding, has also been reported on tasks measuring inhibition in schizophrenia (Huddy et al., 2009). Therefore, patients may be sensitive to reward contingencies and able to speed responses based on reinforcement, despite an impaired ability to differentiate high- and low-probability stimuli explicitly.

It is also surprising that relatively intact implicit adaptive salience was detected in TRS patients despite their taking high doses of antipsychotic medication. A possible explanation relates to the medications taken by the TRS patients: most were taking atypical antipsychotics (nearly half were taking clozapine), which have a relatively lower affinity for dopamine receptors than typical antipsychotics. Interestingly, recent studies suggest that atypical antipsychotics may even normalise reward processing (Nielsen et al., 2012, Smieskova et al., 2015); other studies have found that typical antipsychotics induce greater blunting of striatal responses than atypical antipsychotics (Kirsch et al., 2007). However, it should be noted that the majority of patients in our earlier study (Roiser et al., 2009), in which implicit adaptive salience was disrupted, were also taking atypical antipsychotics; arguing against the notion that differences in medication underlie the different results found between these two studies on the implicit adaptive salience measure.

In relation to our first hypothesis, we observed elevated implicit aberrant salience on the first block only, which may reflect aberrant reward learning, but no group differences in explicit aberrant salience. When digit span (forward) was included as a covariate in the analysis the group*block interaction was no longer significant, although the main effect of group remained. Consistent with the former result, in one previous study we found that this measure correlated with schizotypy in healthy volunteers (Schmidt and Roiser, 2009). However, in our previous studies this measure has not been sensitive to the presence of psychotic symptoms. The explanation for this result is unclear, and therefore we advise that this result is treated with caution until replicated.

The persistence of delusions despite antipsychotic medication in this TRS sample, and the similar explicit aberrant salience scores between the groups, may point to an alternative mechanism than aberrant salience underlying the maintenance of abnormal beliefs. Possibly, dopamine-driven aberrant salience could operate in the early phases of psychosis, but some patients may not be able to unlearn previously learned associations, and thus delusions may be maintained by non-dopaminergic mechanisms in TRS patients. Consistent with this notion, a recent study reported that dopamine synthesis, which is reliably elevated in schizophrenia and its prodrome (Howes et al., 2009), was normal in patients who had not responded to antipsychotic medication and remained psychotic (Demjaha et al., 2012). Another possibility is that there may exist other cognitive distortions that precipitate the expression of delusions other than aberrant salience that we did not test here, for example the “jumping-to-conclusions” bias (Garety et al., 2007) or latent inhibition (Gal et al., 2005, Gray et al., 1995). These surprising results also have some potential implications for treatment. Since this TRS population does not appear to exhibit pronounced aberrant salience, despite the severity of their positive symptoms, in this group it would be worthwhile considering psychosocial interventions that focus more on behavioural or goal-oriented approaches to rehabilitation than on challenging psychotic symptoms. Interestingly, recent evidence supports this suggestion, showing that the degree to which individual inpatient mental health units focus on behavioural or goal-oriented approaches is directly related to the likelihood of successful community discharge (Killaspy et al., 2016).

Several limitations of our study merit comment. First, the small sample size means that the differences between the groups may not be estimated accurately, and therefore we advise that these findings are treated with caution until independently replicated. That said, our a priori power analysis suggested that we did have sufficient power to detect a group difference of the magnitude that we have identified previously on the SAT in similar case–control studies. Second, we did not collect racial or ethnic demographic information from our participants, meaning that we were unable to assess whether the groups were well matched, or representative of the wider population, on these measures. However, in terms of age and gender, our sample was representative of users of mental health rehabilitation services in England (Killaspy et al., 2013). Third, as expected the groups were not matched on working memory ability, which could conceivably affect performance on the SAT. However, including digit span as a covariate in the analyses had little impact on the results. Finally, all TRS patients were taking antipsychotic medication. The aberrant salience hypothesis warrants further investigation in unmedicated patients with delusional schizophrenia. It would also be of great interest to compare patients with schizophrenia who do and do not respond to antipsychotic medication on measures of aberrant salience.

## Conflicts of interest

The authors declare no relevant financial conflicts of interest.

## Funding source

This study was funded by the Wellcome Trust. The funder had no role in the design, conduct or analysis of the study.

Supplementary methods - description of the Salience Attribution Test and statistical analyses. Supplementary methods - description of the Salience Attribution Test and statistical analyses.

Supplementary data to this article can be found online at http://dx.doi.org/10.1016/j.scog.2016.04.002.
