# Supplementary material for: Are persistent delusions in schizophrenia associated with aberrant salience?
Source: Schizophr Res Cogn. 2016 May 18;4:32–8. doi: 10.1016/j.scog.2016.04.002 (PMC4884769; doi:10.1016/j.scog.2016.04.002)
Supplement: Supplementary methods - description of the Salience Attribution Test and statistical analyses. [file mmc1.docx]

**Salience Attribution Test**

The SAT is a speeded reaction time task in which participants respond to a probe (a black square) in order to earn money (34). Money was available on 50% of the trials. The likelihood that money was available on each trial was signalled by a picture (cue) that appeared just before the probe. However, the participants were not informed of the contingencies between the different pictures and reward. Participants were instructed to respond as quickly as they could when the probe appeared on the screen. Participants could earn a maximum of £20, and a minimum of £5, on the test.

Prior to the main test, participants completed a computerised tutorial which featured written instructions, example displays and test trials (34). There were two practice sessions built into the tutorial with the aim of familiarising the participants with the test and providing a baseline measure of their response time (RT). On these practice sessions, a fixation cross appeared at the start of each trial. After a variable interval (minimum 0.5 s, maximum 1.5 s) the probe appeared, and participants responded by pressing a button as quickly as possible. Participants were instructed to try to respond as fast as they were able to, and to do so before the box disappeared. During the first practice session the probe was on the screen for a randomised variable period (minimum duration 0.5 s, maximum duration 1.5 s, mean duration 1 s). One of four feedback messages was displayed on the screen after two seconds - *‘Good’* if the participant responded on time, i.e., before the box disappeared, *‘Try to respond faster’* if they responded after the box disappeared, *‘Too early’* if they responded before the box appeared, and *‘No key pressed’* if they failed to make a response. On the second practice session the mean probe duration was set to be the mean RT from the first session to ensure participants were responding as quickly as possible and to yoke task difficulty to individual performance. The standard deviation (SD) of the fastest half of the trials (SDF) was also calculated, and was used to set the minimum and maximum probe durations for the second practice session (mean from first practice session ±2 × SDF). For the main test, the mean, minimum and maximum probe durations were calculated from the second practice session in the same way. Monetary reinforcement was not provided during the practice sessions.

On the main test participants completed two blocks of 64 trials, where money was available on 50% of trials. On each trial, the likelihood that money was available was signalled by one of four cues that appeared at the top and bottom of the screen just before the onset of the probe (the black square). Four different types of cues were used: red animals; blue animals; red household objects and blue household objects, which varied across two dimensions - colour (blue or red) and ‘form’ (animal or household object). One of the dimensions (for example, colour) was *task-relevant* so that one level of the dimension (for example blue) was reinforced on 28 out of 32 (87.5%) of the trials, while only 4 out of 32 (12.5%) trials of the other level were reinforced. For example, if ‘colour’ was the reinforced dimension with blue as high probability, 14 out of 16 blue animals and 14 out of 16 blue household objects would be reinforced, compared with only 2 out of 16 red animals and 2 out of 16 red household objects. The other dimension, in this example ‘form’, was *task-irrelevant*, so that 16 out of 32 (50%) of both levels (animals and household objects) were reinforced.

At the beginning of each trial a fixation cross appeared. After 1000 ms, while the fixation cross remained on-screen, one of the four cues was displayed at the top and bottom of the screen and remained on-screen until the end of the trial. After a variable period of time (between 0.5 and 1.5 s) the probe appeared and participants attempted to respond before it disappeared. The probe duration was calculated according to the participant's responses on the second practice block, as described above. After 2.25 s, auditory and visual feedback was presented for 1.5 s. Four different versions of the SAT were used, each with a different stimulus feature (blue, red, animal or household object) reinforced with high probability. Each participant was administered the same version (i.e. with identical cue-reinforcement contingencies) for both blocks of the SAT.

If the trial was not reinforced, the message *‘Sorry – no money available’* was displayed. If the trial was reinforced, participants won between 5 and 100 pence, depending on the latency of their response. On reinforced trials where participants either made no response or responded after the probe had disappeared, the message *‘Missed: 5 pence’* was displayed. If participants responded prematurely (<100 ms after the onset of the probe), the message displayed was *‘Too early: 5 pence’*. On reinforced trials where participants responded before the probe disappeared, but slower than their mean RT, the message *‘Hit – good: 10 pence’* was displayed. If participants responded faster than their mean RT, the message *‘Quick – very good: X pence’* was displayed (for responses up to 1.5 SDFs faster than their mean RT) and *‘Very quick – excellent: X pence’* (for responses faster than their mean RT by at least 1.5 SDFs). The reward was scaled according to X=10+90×(mean RT – trial RT)/(3×SDF), up to a maximum of 100 pence. For example, a response one SDF faster than the mean was reinforced with 40 pence, a response two SDFs faster was reinforced with 70 pence, and any responses three SDFs or faster than the mean were reinforced with 100 pence. The money won on each trial was added to the participant's running total for that block, Y, which was displayed underneath the feedback: *‘Total – £Y’*. On reinforced trials, a 0.5 s tone sounded, frequency: (300+(10×X)) Hz. At the end of each block, participants indicated, using a visual analogue scale (VAS), their estimate (percentage) of the reinforcement probabilities for each of the four different cues.

Four measures of motivational salience were calculated for each block. *Adaptive salience* was defined in two ways: implicit adaptive salience was defined as the speeding of responses on high probability reinforcement trials relative to low probability reinforcement trials (i.e. the average of the two low-probability stimulus classes – red animals and red household objects in the above example - minus the average of the two high-probability stimulus classes – blue animals and blue household objects in the above example); explicit adaptive salience was defined as the increase in probability rating for high probability reinforcement trials relative to low probability reinforcement trials (the average of the two high-probability stimulus classes (blue in the above example) minus the average of the two low-probability stimulus classes (red in the above example)). *Aberrant salience* was similarly defined in two ways, as the absolute difference in RT (implicit) or VAS rating (explicit) between the two levels of the task-irrelevant stimulus dimension (the difference between animals and household objects in the above example). The number of premature responses and omissions were also recorded for each stimulus type on each block.

## Statistical analysis

The data were analysed using the Statistical Package for Social Sciences, version 16 (SPSS Inc., Chicago, IL, USA). Demographic measures were analysed using independent samples *t*-tests and chi-squared tests. SAT and digit-span data were analysed using repeated measures analysis of variance. For digit span, stage (forwards/backwards) was the within-subjects variable, while for SAT, block (1/2) and probability (high/low) were the within-subjects variables. Group (patient/control) was the between subjects variable on both analyses. Initially, gender was included as an additional between subjects variable for all analyses, but in no instance was the main effect of gender or any interaction significant; therefore gender was dropped from all models. Post-hoc analyses were conducted using t-tests where a significant interaction term was identified. Implicit aberrant salience data were log transformed prior to analysis to reduce skew. For clarity untransformed values are presented in the tables and figures. Omission error data were analysed using a Mann-Whitney U test since the data were not normally distributed, even after log transformation. For all analyses *P*<0.05 was considered significant while 0.05<*P*<0.1 was considered a trend towards significance. Correlations with O-LIFE subscales and clinical variables were performed using Pearson’s *r.* Due to the high number of tests performed, a more stringent value of *P*<0.01 was adopted for significance in the correlation analyses, with 0.01<*P*<0.05 considered a trend towards significance.
